# New Staphylinidae (Coleoptera) records with new collection data from New Brunswick and eastern Canada: Tachyporinae

**DOI:** 10.3897/zookeys.186.2491

**Published:** 2012-04-26

**Authors:** Reginald P. Webster, Jon D. Sweeney, Ian DeMerchant

**Affiliations:** 1 Natural Resources Canada, Canadian Forest Service - Atlantic Forestry Centre, 1350 Regent St., P.O. Box 4000, Fredericton, NB, Canada E3B 5P7

**Keywords:** Staphylinidae, Tachyporinae, new records, Canada, New Brunswick

## Abstract

Twenty-three species of Tachyporinae are newly recorded from New Brunswick. This brings the total number of Tachyporinae known from the province to 70. *Lordithon campbelli* Schülke is newly recorded for Canada and we provide the first documented records of *Tachinus addendus* Horn and *Tachinus frigidus* Erichson for New Brunswick. Collection and habitat data are presented and discussed for each species. A list of Tachyporinae species currently known from the province of New Brunswick is presented.

## Introduction

Intensive collecting of rove beetles (family Staphylinidae) in New Brunswick by the first author since 2003 and records from by-catch samples obtained during a study to develop a general attractant for the detection of invasive species of Cerambycidae have yielded many new provincial and national records. These are being published in a series of papers, each focusing on one or more subfamilies. This paper treats the subfamily Tachyporinae. The Tachyporinae of Canada and North America are fairly well known taxonomically thanks to various revisions by J.M. Campbell; *Tachinus* (Campbell 1973, 1975b, 1988), *Coproporus* and *Cilea* (Campbell 1975a), *Sepedophilus* (Campbell 1976), *Tachyporus* (Campbell 1979), *Carphacis* (Campbell 1980), *Lordithon* (Campbell 1982), *Mycetoporus* and *Ischnosoma* (Campbell 1991), *Nitidotachinus* (Campbell 1993a), and *Bryoporus* and *Bryophacis* (Campbell 1993b).

Tachyporinae can be found in a wide variety of habitats. *Tachinus* species are often found in decaying organic materials such as dung, rotting mushrooms, carrion, and compost, although some species are found in leaf litter and moist debris near streams, e.g., *Tachinus limbatus* Melsheimer (Campbell 1973). *Nitidotachinus* species are usually found in leaf litter or moss near streams, often in cool, shaded sites (Campbell 1993a). *Tachyporus*, *Mycetoporus*, *Bryoporus*, *Bryophacis*, and *Ischnosoma* species are usually associated with various kinds of litter and moss in forests and various wetland types, depending on the species (Campbell 1979, 1991, 1993b). *Tachyporus* species are often swept from vegetation in fields and other open habitats (Campbell 1979). Larvae and adults of *Lordithon* are associated with mushrooms and are active predators of Diptera larvae (Campbell 1982). Some *Sepedophilus* species are found in rotting wood, under loose bark, and in decaying and often moldy organic materials, such as rotting leaves (Campbell 1976); others are associated with polypore fungi or fleshy fungi on trees, depending on the species (Newton et al. 2000). Some species may be mycetophagous (Newton et al. 2000). Our only *Coproporus* species is subcortical (Campbell 1975b). However, in general, little is known about the biology of our North American Tachyporinae.

Thirty-six species of Tachyporinae were reported from New Brunswick by Campbell and Davies (1991). Nine species were added to the faunal list in revisions by Campbell (1991, 1993a, b) and from general surveys by Klimaszewski et al. (2005) and Majka and Klimaszewski (2008). Majka et al. (2011) reported *Tachinus addendus* Horn and *Tachinus frigidus* Erichson as occurring in New Brunswick but did provide any supporting references or data for the records. Here, we report an additional 23 species, bringing the total number of Tachyporinae known from New Brunswick to 70.

## Methods and conventions

The following records are based on specimens collected as part of a general survey by the first author to document the Coleoptera fauna of New Brunswick and from by-catch samples obtained during a study to develop a general attractant for the detection of invasive species of Cerambycidae.

### Collection methods

Various collection methods were employed to collect the Tachyporinae reported in this study. Details are outlined in Campbell (1973) and Webster et al. (2009, Appendix). See Webster et al. (2012) for details of the methods used for deployment of Lindgren 12-funnel traps and sample collection. A description of the habitat was recorded for all specimens collected during this survey. Locality and habitat data are presented exactly as on labels for each record. This information, as well as additional collecting notes, is summarized and discussed in the collection and habitat data section for each species.

### Specimen preparation

Examples of males of most species were dissected to confirm their identity. The genital structures were dehydrated in absolute alcohol and mounted in Canada balsam on celluloid microslides and pinned with the specimens from which they originated.

### Distribution

Distribution maps, created using ArcMap and ArcGIS, are presented for each species in New Brunswick. Every species is cited with current distribution in Canada and Alaska, using abbreviations for the state, provinces, and territories. New provincial records are indicated in bold under Distribution in Canada and Alaska. The following abbreviations are used in the text:

**Table:** 

**AK**	Alaska	**MB**	Manitoba
**YT**	Yukon Territory	**ON**	Ontario
**NT**	Northwest Territories	**QC**	Quebec
**NU**	Nunavut	**NB**	New Brunswick
**BC**	British Columbia	**PE**	Prince Edward Island
**AB**	Alberta	**NS**	Nova Scotia
**SK**	Saskatchewan	**NF & LB**	Newfoundland and Labrador*

* Newfoundland and Labrador are each treated separately under the current Distribution in Canada and Alaska.

Acronyms of collections examined and referred to in this study are as follows:

AFCAtlantic Forestry Centre, Natural Resources Canada, Canadian Forest Service, Fredericton, New Brunswick, Canada


CNCCanadian National Collection of Insects, Arachnids and Nematodes, Agriculture and Agri-Food Canada, Ottawa, Ontario


NBMNew Brunswick Museum, Saint John, New Brunswick, Canada


RWCReginald P. Webster Collection, Charters Settlement, New Brunswick, Canada


## Results

Twenty-three species of Tachyporinae are newly recorded from New Brunswick. Twelve of these are newly recorded from the Maritime provinces (New Brunswick, Nova Scotia, Prince Edward Island) of Canada, including *Lordithon campbelli* Schülke, which is newly recorded for Canada. The first documented records of *Tachinus addendus* and *Tachinus frigidus* from New Brunswick are provided. This brings the total number of species known from New Brunswick to 70 (Table 1).

**Table 1.** Species of Tachyporinae (Staphylinidae) recorded from New Brunswick, Canada.

Subfamily Tachyporinae MacLeay
Tribe Tachyporini MacLeay
*Cilea silphoides* (Linnaeus)
*Coproporus ventriculus* (Say)
*Nitidotachinus scrutator* (Gemminger & Harold)
*Nitidotachinus tachyporoides* (Horn)
*Nitidotachinus horni* (Campbell)*
*Sepedophilus cinctulus* (Erichson)*
*Sepedophilus crassus* (Gravenhorst)*
*Sepedophilus littoreus* (Linnaeus)
*Sepedophilus marshami* (Stephens)
*Sepedophilus occultus* Casey**
*Sepedophilus testaceus* (Fabricius)
*Sepedophilus versicolor* (Casey)**
*Tachinus addendus* Horn
*Tachinus basalis* Erichson
*Tachinus canadensis* Horn**
*Tachinus corticinus* Gravenhorst
*Tachinus fimbriatus* Gravenhorst*
*Tachinus fumipennis* (Say)
*Tachinus limbatus* Melsheimer
*Tachinus luridus* Erichson
*Tachinus frigidus* Erichson
*Tachinus memnonius* Gravenhorst
*Tachinus picipes* Erichson
*Tachinus quebecensis* Robert
*Tachinus rufipes* (DeGeer)
*Tachinus schwarzi* Horn*
*Tachinus vergatus* Campbell**
*Tachinus thruppi* Hatch
*Tachyporus abdominalis* (Fabricius)
*Tachyporus browni* Campbell
*Tachyporus canadensis* Campbell
*Tachyporus dispar* (Paykull)
*Tachyporus flavipennis* Campbell
*Tachyporus inornatus* Campbell
*Tachyporus lecontei* Campbell**
*Tachyporus maculicollis* LeConte*
*Tachyporus nanus* Erichson**
*Tachyporus nimbicola* Campbell
*Tachyporus nitidulus* (Fabricius)
*Tachyporus pulchrus* Blatchley**
*Tachyporus rulomoides* Campbell
*Tachyporus transversalis* Gravenhorst**
Tribe Mycetoporini Thomson
*Bryophacis smetanai* Campbell
*Bryoporus rufescens* LeConte
*Bryoporus testaceus* LeConte
*Carphasis nepigonensis* (Bernhauer)
*Ischnosoma fimbriatum* Campbell
*Ischnosoma flavicolle* (LeConte)**
*Ischnosoma pictum* (Horn)
*Ischnosoma splendidum* (Gravenhorst)*
*Ischnosoma virginicum* (Bernhauer)
*Lordithon (Bolitobius) fungicola* Campbell
*Lordithon (Bolitobius) kellyi* Malkin
*Lordithon (Bolitobius) longiceps* (LeConte)*
*Lordithon (Bolitobius) quaesitor* (Horn)*
*Lordithon (Lordithon) anticus* (Horn)
*Lordithon (Lordithon) appalachianus* Campbell
*Lordithon (Lordithon) axillaris* (Gravenhorst)**
*Lordithon (Lordithon) campbelli* Schülke***
*Lordithon (Lordithon) facilis* (Casey)
*Lordithon (Lordithon) niger* (Gravenhorst)**
*Lordithon (Lordithon) scutellaris* Campbell
*Lordithon (Lordithon) thoracicus thoracicus* (Fabricius)
*Mycetoporus americanus* Erichson**
*Mycetoporus consors* LeConte
*Mycetoporus horni* (Bernhauer & Schubert)
*Mycetoporus inquisitus* Casey
*Mycetoporus lucidulus* LeConte
*Mycetoporus rugosus* Hatch*
*Mycetoporus triangulatus* Campbell

**Notes:** *New to province, **New to Maritime provinces, ***New to Canada.

## Species accounts

All species below are newly recorded for New Brunswick, Canada. Species followed by ** are newly recorded from the Maritime provinces; species followed by *** are newly recorded for Canada.

The classification of the Tachyporinae follows Bouchard et al. (2011).

### Family Staphylinidae Latreille, 1802

Subfamily Tachyporinae MacLeay, 1825

Tribe Tachyporini MacLeay, 1825

#### Nitidotachinus horni

Campbell, 1973

http://species-id.net/wiki/Nitidotachinus_horni

Map 1

##### Material examined.

**New Brunswick, Albert Co.**, Caledonia Gorge P.N.A. (Protected Natural Area), at Canada Creek, 45.7808°N, 64.7775°W, 4.VII.2011, R. P. Webster, cold, clear, and shaded rocky brook in mixed forest, in saturated moss (1, NBM). **Carleton Co.**, Jackson Falls, Bell Forest, 46.2208°N, 67.7231°W, 2.VI.2005, R. P. Webster, mature hardwood forest, in litter on margin of cold spring-fed brook (1, RWC); Meduxnekeag Valley Nature Preserve, 46.1895°N, 67.6704°W, 13.VI.2010, 18.VI.2010, R. P. Webster, hardwood forest, margin of cold shaded spring-fed brook, under small rocks and in gravel (6, RWC).

**Map 1.**
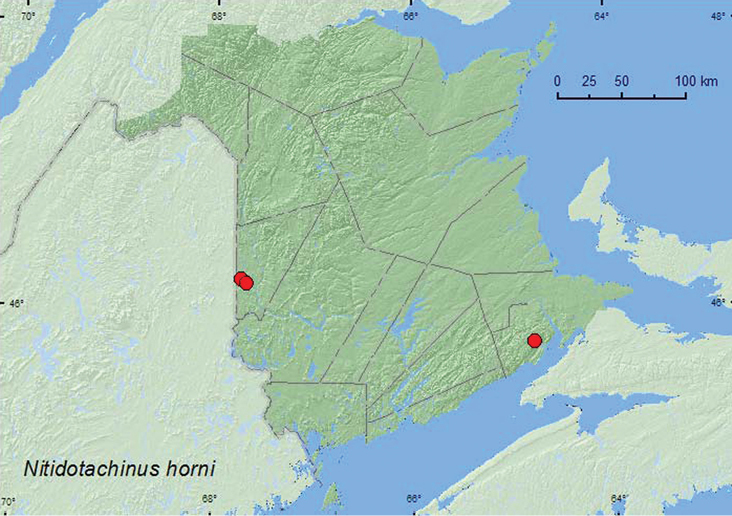
Collection localities in New Brunswick, Canada of *Nitidotachinus horni*.

##### Collection and habitat data.

Adults of this rarely collected species were found in seepage areas, under stones on a stream margin, an alder thicket, and forest litter (Campbell 1988). In New Brunswick, adults were collected from under small rocks, in gravel, or in litter and moss along the margins of cold, shaded, spring-fed brooks in hardwood forests. Adults were collected during June and July.

##### Distribution in Canada and Alaska.

ON, QC, **NB**, NS (Campbell 1973, 1988).

#### Sepedophilus cinctulus

(Erichson, 1839)

http://species-id.net/wiki/Sepedophilus_cinctulus

Map 2

##### Material examined.

**New Brunswick, Albert Co.**, Caledonia Gorge P.N.A., 45.8257°N, 64.7791°W, 6.VII.2011, R. P. Webster, old hardwood forest (sugar maple and beech), on *Polyporus varius* (1, NBM); Caledonia Gorge P.N.A., near Turtle Creek, 45.8380°N, 64.8484°W, 3.VII.2011, A. Fairweather & R. P. Webster, old-growth sugar maple and yellow birch forest, on *Polyporus varius* (1, NBM). **Carleton Co.**, Richmond, near Hovey Hill P.N.A. (Protected Natural Area), 46.1155°N, 67.7631°W, 24.V.2005, R. P. Webster, clear-cut, in well rotted log (1, NBM); Jackson Falls, Bell Forest, 46.2200°N, 67.7231°W, 16.IX.2006, R. P. Webster, mature hardwood forest, on fleshy polypore fungi on beech log (8 ♂, 7 ♀, NBM, RWC); same locality and forest type, 23–28.IV.2009, 14–20.V.2009, 20–26.V.2009, 8–16.VI.2009, R. Webster, V. Webster, & M.-A. Giguère, Lindgren funnel traps (4, AFC). **Queens Co.**, near Queenstown, 45.6904°N, 66.1455°W, 13.V.2008, R. P. Webster, old growth hardwood forest, under bark of sugar maple (1, NBM); Cranberry Lake P.N.A., 46.1125°N, 65.6075°W, 5–12.V.2009, 10–15.VII.2009, R. Webster & M.-A. Giguère, mature red oak forest, Lindgren funnel traps (2, AFC, RWC). **Sunbury Co.**, Acadia Research Forest, 46.0188°N, 66.3765°W, 17.VIII.2007, R. P. Webster, mature red spruce and red maple forest, in *Piptoporus betulinus* (birch polypore) (1, AFC); Acadia Research Forest, 45.9866°N, 66.3841°W, 19–25.V.2009, R. Webster & M.-A. Giguère, mature (110 year-old) red spruce forest with scattered red maple and balsam fir, Lindgren funnel trap (1, AFC). **York Co.**, 15 km W of Tracy off Rt. 645, 45.6848°N, 66.8821°W, 9.V.2007, R. P. Webster, old red pine forest, under bark of log (1, NBM); same locality and forest type but 11–19.V.2009, 19–25.V.2009, R. Webster & M.-A. Giguère, Lindgren funnel traps (2, AFC); 14 km WSW of Tracy, S of Rt. 645, 45.6741°N, 66.8661°W, 26.IV-10.V.2009, R. Webster & C. MacKay, old mixed forest with red and white spruce, red and white pine, balsam fir, eastern white cedar, red maple, and *Populus* sp., Lindgren funnel trap (1, AFC).

**Map 2.**
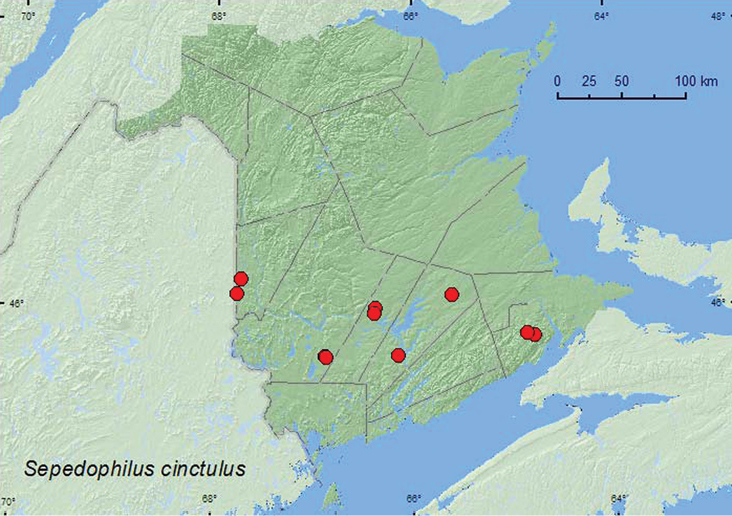
Collection localities in New Brunswick, Canada of *Sepedophilus cinctulus*.

##### Collection and habitat data.

Campbell (1976) reported that most specimens of this species were collected from under bark. Others were found in mushrooms, *Polyporus betulinus* (Bull.) Fr., on dead logs, dead beech (*Fagus grandifolia* Ehrh.), and tree trunks and in tree holes. In New Brunswick, specimens were found on fleshy polypore fungi on a beech log, in *Piptoporus betulinus* (Bull.) P. Karst. (birch polypore), on *Polyporus varius* Fr., and in a well-rotted log. This species was commonly collected in Lindgren funnel traps in various forest types; mature hardwood forests with sugar maple (*Acer saccharum* Marsh.) and beech, old red oak (*Quercus rubra* L.) forest, old-growth hardwood forest with sugar maple and yellow birch (*Betula alleghaniensis* Britt.), 110-year-old red spruce (*Picea rubens* Sarg.) forest with red maple (*Acer rubrum* L.), old red pine (*Pinus resinosa* Ait.) forest, and an old mixed forest. Adults were collected during April, May, June, July, August, and September.

##### Distribution in Canada and Alaska.

ON, QC, **NB**, NS (Campbell 1976; Bishop et al. 2009).

#### Sepedophilus crassus

(Gravenhorst, 1802)

http://species-id.net/wiki/Sepedophilus_crassus

Map 3

##### Material examined.

**New Brunswick, Albert Co.**, Caledonia Gorge P.N.A., 45.8257°N, 64.7791°W, 6.VII.2011, R. P. Webster, old hardwood forest (sugar maple and beech), on *Polyporus varius* (2, NBM). **Carleton Co.**, Meduxnekeag Valley Nature Preserve, 46.1907°N, 67.6740°W, 4.VIII.2006, 8.VIII.2006, R. P. Webster, hardwood forest, in fleshy polypore fungi on side of log (2, NBM); Jackson Falls, Bell Forest, 46.2200°N, 67.7231°W, 16.IX.2006, R. P. Webster, mature hardwood forest, on fleshy polypore fungi on beech log (1 ♂, RWC); same locality, collector and forest type, 7.VI.2007, in polypore fungi on large basswood log (1, NBM); same locality and forest type, 31.VII-7.VIII.2009, 7–12.VIII.2009, R. Webster & M.-A. Giguère, Lindgren funnel traps (2, AFC). **Sunbury Co.**, Acadia Research Forest, 45.9866°N, 66.3841°W, 30.VI-8.VII.2009, 4–11.VIII.2009, R. Webster & M.-A. Giguère, mature (110 year-old) red spruce forest with scattered red maple and balsam fir, Lindgren funnel traps (2, AFC). **York Co.**, Fredericton, Odell Park, 45.9570°N, 66.6695°W, 19.VI.2005, R. P. Webster, old growth hemlock forest, on bracket fungi (6 ♂, 6 ♀, NBM, RWC); Charters Settlement, 45.8286°N, 66.7365°W, 15.IX.2006, R. P. Webster, mature mixed forest, in polypore fungi on dead (standing) spruce (1 ♀, RWC).

**Map 3.**
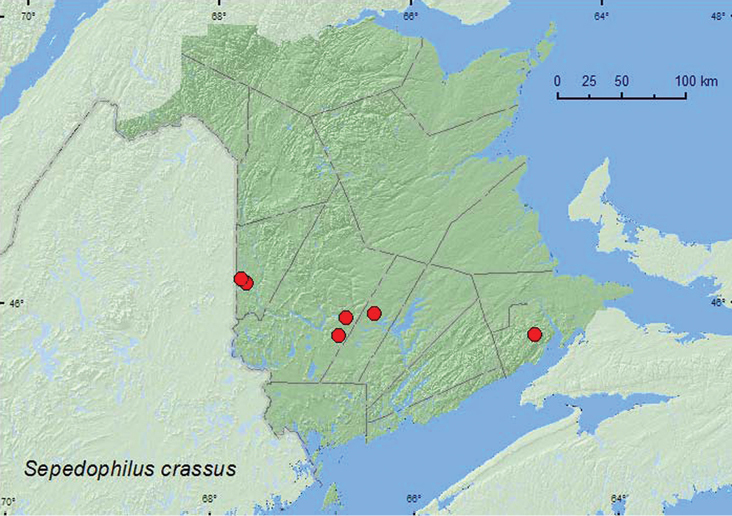
Collection localities in New Brunswick, Canada of *Sepedophilus crassus*.

##### Collection and habitat data.

Campbell (1976) reported that adults were frequently collected from rotten wood, from deep layers of decaying leaves, and from bracket fungi and mushrooms. Most specimens from New Brunswick were collected from fleshy polypore fungi and bracket fungi on standing dead trees and logs. Some adults were also collected from Lindgren funnel trap samples. Two specimens were collected from *Polyporus varius* Fr. on a rotten log. This species was found in sugar maple and beech forests, a red spruce forest, an old-growth hemlock (*Tsuga canadensis* (L.)) forest, and mixed forests. Adults were collected during June, July, August, and September.

##### Distribution in Canada and Alaska.

ON, QC, **NB**, NS (Campbell 1976; Bishop et al. 2009).

#### Sepedophilus occultus

(Casey, 1884)**

http://species-id.net/wiki/Sepedophilus_occultus

Map 4

##### Material examined.

**CANADA, New Brunswick, Gloucester Co.**, near Black Rock, 47.7411°N, 65.2577°W, 8.VI.2006, R. P. Webster, old growth eastern white cedar swamp, inside well rotted fungus covered log (5 ♂, 4 ♀, NBM, RWC). **York Co.** Charters Settlement, 45.8395°N, 66.7391°W, 22.VIII.2005, R. P. Webster, mixed forest, in well rotted fungus covered log (1 ♂, NBM); same locality and collector but 45.8286°N, 66.7365°W, 24.VI.2006, mature mixed forest, in polypore fungi on dead standing *Populus* sp. (1 ♂, RWC).

**Map 4.**
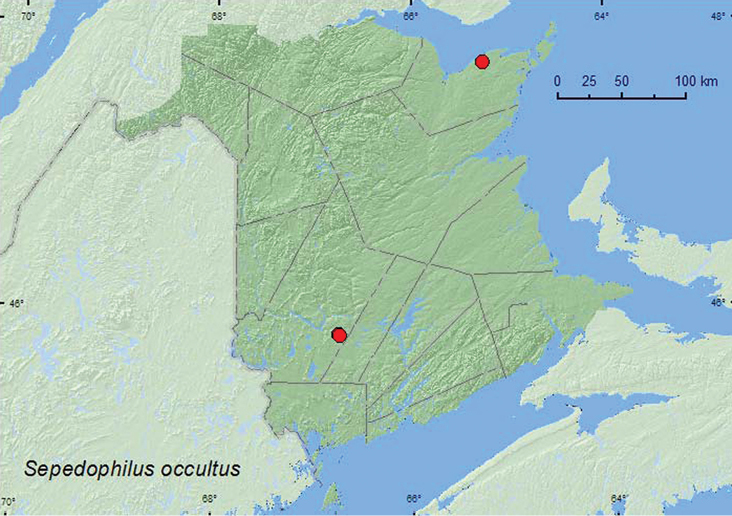
Collection localities in New Brunswick, Canada of *Sepedophilus occultus*.

##### Collection and habitat data.

In the United States, this species has been collected from under bark, under a brush pile, and by sifting humus (Campbell 1976). The New Brunswick specimens were collected from the inside of well-rotted, fungus-covered logs and from polypore fungi on dead, standing *Populus* sp. This species was found in an old-growth eastern white cedar (*Thuja occidentalis* L.) swamp and in mature to old mixed forests. Adults were collected during June and August.

##### Distribution in Canada and Alaska.

ON, QC, **NB** (Paquin and Dupérré 2001; Brunke and Marshall 2011).

#### Sepedophilus versicolor

(Casey, 1884)**

http://species-id.net/wiki/Sepedophilus_versicolor

Map 5

##### Material examined.

**CANADA, New Brunswick, Queens Co.**, Grand Lake near Scotchtown, 45.8762°N, 66.1816°W, 25.V.2006, R. P. Webster, oak and maple forest, under bark of red oak (1 ♀, RWC); same locality, forest type and collector, 19.IX.2006, on fleshy polypore fungi (1 ♂, 2 ♀, RWC); Grand Lake Meadows P.N.A., 45.8227°N, 66.1209°W, 15–29.VI.2010, R. Webster & C. MacKay, old silver maple forest with green ash and seasonally flooded marsh, Lindgren funnel trap (1, AFC); same locality data and forest type, 5–17.VIII.2011, 17–30.VIII.2011, M. Roy & V. Webster, Lindgren funnel traps (2, AFC, NBM). **Sunbury Co.**, Burton, near Sunpoke Lake, 45.7665°N, 66.5545°W, 15.V.2004, R. P. Webster, red oak and red maple forest with scattered white pine, under bark (1 ♀, RWC); Lakeville Corner, 45.9007°N, 66.2423°W, 27.VIII.2006, R. P. Webster, silver maple swamp, on polypore fungi on *Populus* sp. log (2 ♂, RWC). **York Co.**, Charters Settlement, 45.8395°N, 66.7391°W, 5.IX.2006, R. P. Webster, mixed forest, among decaying (moldy) corncobs and cornhusks (1 ♀, RWC).

**Map 5.**
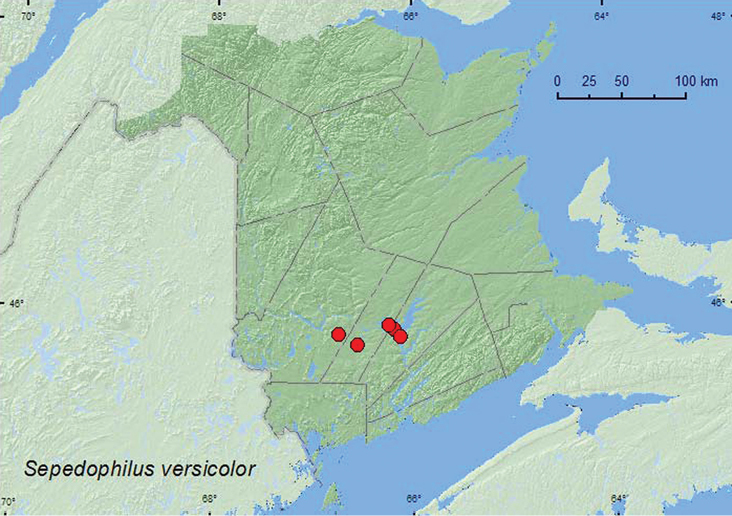
Collection localities in New Brunswick, Canada of *Sepedophilus versicolor*.

##### Collection and habitat data.

Campbell (1976) reported this species from mushrooms. In New Brunswick, specimens were collected from polypore fungi on logs, from under bark, and among moldy corncobs and cornhusks. This species was found in red oak and red maple forests, old silver maple (*Acer saccharinum* L.) forests, and near a mixed forest. Adults were collected during May, August, and September.

##### Distribution in Canada and Alaska.

ON, **NB**. (Brunke and Marshall 2011). Campbell (1976) did not report this species from Canada. However, there are two specimens of this species in the Canadian National Collection from Turkey Point, Ontario collected in 1975 that first establish this species as a member of the Canadian fauna. Brunke and Marshall (2011) reported an additional record from Rondeau Provincial Park, Ontario. In the United States, this species occurs from New Hampshire west to Iowa and south to Florida (Campbell 1976).

#### Tachinus addendus

Horn, 1877

http://species-id.net/wiki/Tachinus_addendus

Map 6

##### Material examined.

**Additional New Brunswick records, Albert Co.**, Shepody N.W.A., Mary’s Point Section, 45.7260°N, 64.6640°W, 12.IX.2004, R. P. Webster, spruce forest, in decaying fleshy fungi (1, RWC); Caledonia Gorge P.N.A., near Turtle Creek, 45.8380°N, 64.8484°W, 3.VII.2011, R. P. Webster, old-growth sugar maple and yellow birch forest, in moose dung (1, NBM). **Carleton Co.**, Meduxnekeag River Valley Nature Preserve, 46.1907°N, 67.6740°W, 23.VI.2006, 7.IX.2004, R. P. Webster, mature hardwood forest,in rotting mushrooms (8, NBM, RWC); Two Mile Brook Fen, 46.3702°N, 67.6772°W, 4.VIII.2006, R. P. Webster, mixed forest, in gilled mushroom (1, NBM). **Queens Co.**, Cranberry Lake P.N.A, (Protected Natural Area) 46.1125°N, 65.6075°W, 2.IX.2009, R. Webster & M.-A. Giguère, old red oak forest, in decaying gilled mushroom (1, AFC). **Restigouche Co.**, Mount Carleton Provincial Park, Mt. Sagamook, 2000 ft. elev., 47.4112°N, 66.8599°W, 2.IX.2006, R. P. Webster, mixed forest, in decaying gilled mushroom (1, NBM); Jacquet River Gorge P.N.A., 47.8160°N, 66.0083°W, 14.VIII.2010, R. P. Webster, old eastern white cedar forest, in decaying mushrooms (1, NBM); Dionne Brook P.N.A., 47.9064°N, 68.3441°W, 23.VIII–19.IX.2011, M. Roy & V. Webster, old-growth white spruce and balsam fir forest, Lindgren funnel trap (1, NBM). **Saint John Co.**, Dipper Harbour, 45.1169°N, 66.3771°W, 15.V.2006, R. P. Webster, upper margin of sea beach, in decaying sea wrack under alders (1, RWC). **York Co.**, Browns Mountain Fen, 45.8965°N, 67.6344°W, 5.VIII.2004, J. Edsall & R. Webster, mixed forest, in decaying fleshy fungi (2, NBM, RWC).

**Map 6.**
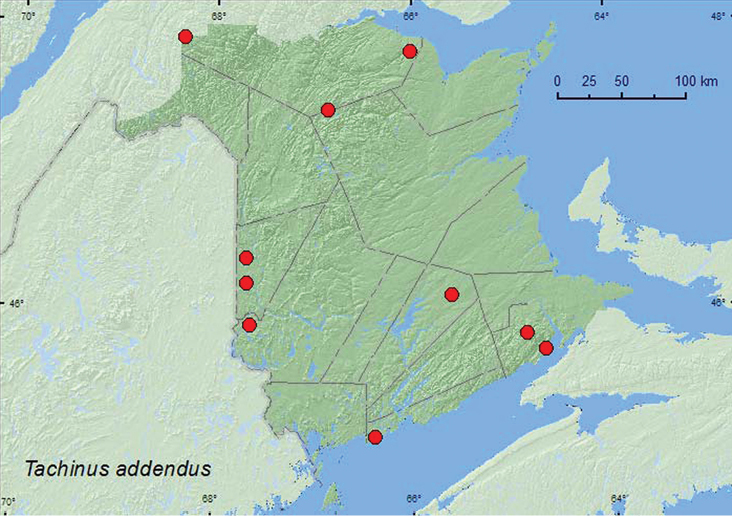
Collection localities in New Brunswick, Canada of *Tachinus addendus*.

##### Collection and habitat data.

This species has been collected from dung, rotting mushrooms, deciduous leaf litter, and pine duff (Campbell 1973). Most adults from New Brunswick were collected from decaying mushrooms in hardwood and mixed forests. One individual was collected from decaying sea wrack under alders (*Alnus* sp.) on the upper margin of a sea beach, another was found in moose dung. Adults were collected during May, June, July, August, and September.

##### Distribution in Canada and Alaska.

MB, ON, QC, NB, NS (Campbell 1973, 1988). *Tachinus addendus* was listed as occurring in New Brunswick by Majka et al. (2011) without any supporting references or data. Here, we provide the first documented records from New Brunswick.

#### Tachinus canadensis

Horn, 1877**

http://species-id.net/wiki/Tachinus_canadensis

Map 7

##### Material examined.

**New Brunswick, Sunbury Co.**, Lakeville Corner, 45.9007°N, 66.2423°W, 10.IX.2006, R. P. Webster, silver maple forest on ridge with red oaks, on gilled mushrooms (2, RWC).

**Map 7.**
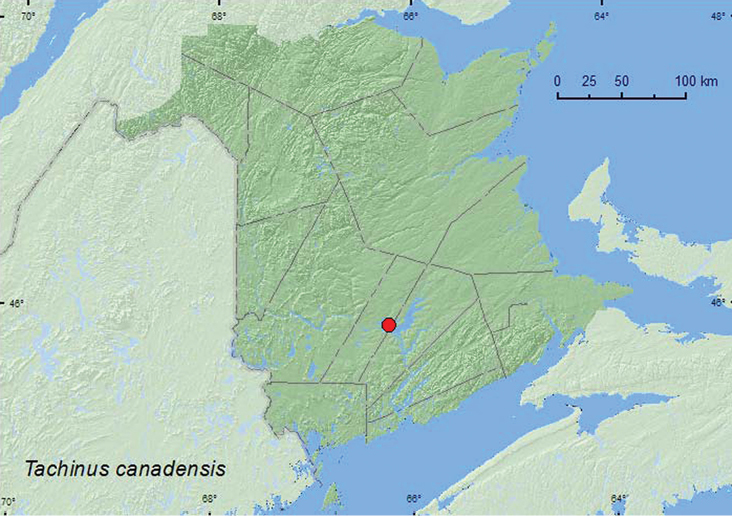
Collection localities in New Brunswick, Canada of *Tachinus canadensis*.

##### Collection and habitat data.

Little was previously known about the habitat associations of this species other than some specimens having been collected from mushrooms (Campbell 1973). The two specimens from New Brunswick were collected from gilled mushrooms near a silver maple swamp during September. Campbell (1973) commented that the late period of annual activity (September and October) was quite different from other species occurring in eastern North America.

##### Distribution in Canada and Alaska.

ON, QC, **NB** (Campbell 1973).

#### Tachinus fimbriatus

Gravenhorst, 1802

http://species-id.net/wiki/Tachinus_fimbriatus

Map 8

##### Material examined.

**New Brunswick, Carleton Co.**, Hovey Hill P.N.A., 46.1115°N, 67.7770°W, 7.IX.2004, R. P. Webster, mature mixed forest, in well rotted *Boletus* mushroom (2, RWC).

**Map 8.**
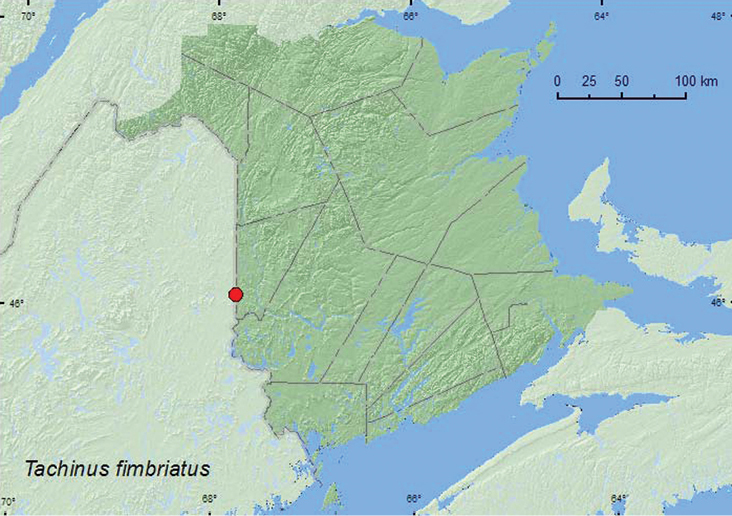
Collection localities in New Brunswick, Canada of *Tachinus fimbriatus*.

##### Collection and habitat data.

*Tachinus fimbriatus* is usually collected from rotting mushrooms (Campbell 1973), as were the two specimens from New Brunswick. Adults were collected in a mature mixed forest during September.

##### Distribution in Canada and Alaska.

ON, QC, **NB,** NS (Campbell 1973, 1988).

#### Tachinus frigidus

Erichson, 1840

http://species-id.net/wiki/Tachinus_frigidus

Map 9

##### Material examined.

**Additional New Brunswick records, Albert Co.**, Shepody N.W.A., Mary’s Point Section, 45.7260°N, 64.6640°W, 12.IX.2004, R. P. Webster, spruce forest, in decaying fleshy fungi (gilled mushroom) (1, RWC).

**Map 9.**
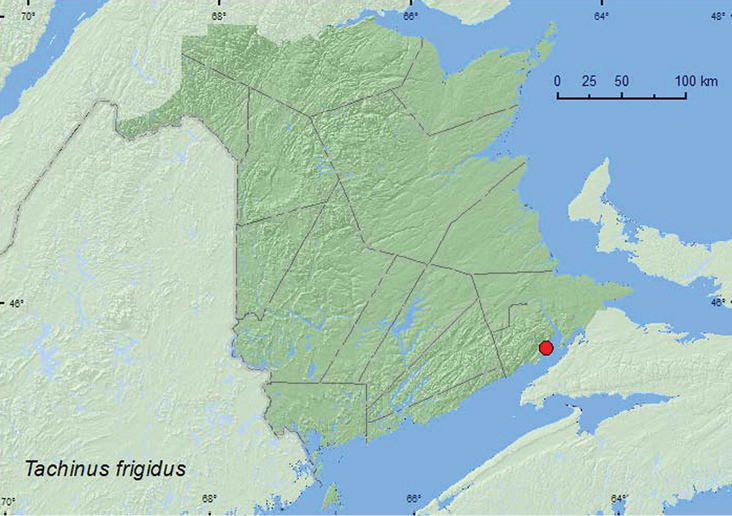
Collection localities in New Brunswick, Canada of *Tachinus frigidus*.

##### Collection and habitat data.

Campbell (1973, 1988) reported that most specimens of this northern transcontinental species were collected from under animal dung or decaying mushrooms. Adults were also collected from the mouth of animal burrows, in leaf litter and other kinds of decaying organic matter. In Alberta, *Tachinus frigidus* was considered to be a mature forest (conifer-dominated) specialist (Pohl et al. (2007). The only specimen from New Brunswick was collected from a decaying fleshy mushroom during September in a mature, coastal red spruce forest.

##### Distribution in Canada and Alaska.

AK, YT, NT, BC, AB, MB, ON, QC, NB, NS, LB (Campbell 1973). *Tachinus frigidus* was listed as occurring in New Brunswick by Majka et al. (2011) without any supporting references or data. Here we provide the first documented records from New Brunswick.

#### Tachinus schwarzi

Horn, 1877

http://species-id.net/wiki/Tachinus_schwarzi

Map 10

##### Material examined.

**New Brunswick, Sunbury Co.**, Acadia Research Forest, 46.0188°N, 66.3796°W, 17.VIII.2007, R. P. Webster, mature red spruce and red maple forest, in decaying fleshy polypore fungi on standing dead spruce (1 ♂, AFC). **Restigouche Co.**, vic. Summit Depot, 47.7836°N, 68.3227°W, 21.VII.2010, M. Turgeon & R. Webster, clear-cut, on decaying *Climacodon septentrionale* on dead (standing) yellow birch (1, RWC); Dionne Brook P.N.A. 47.9030°N, 68.3503°W, 9.VIII.2011, R. P. Webster, old-growth northern hardwood forest, on *Climacodon septentrionale* (Fr.) P. Karst. on sugar maple (2, RWC).

**Map 10.**
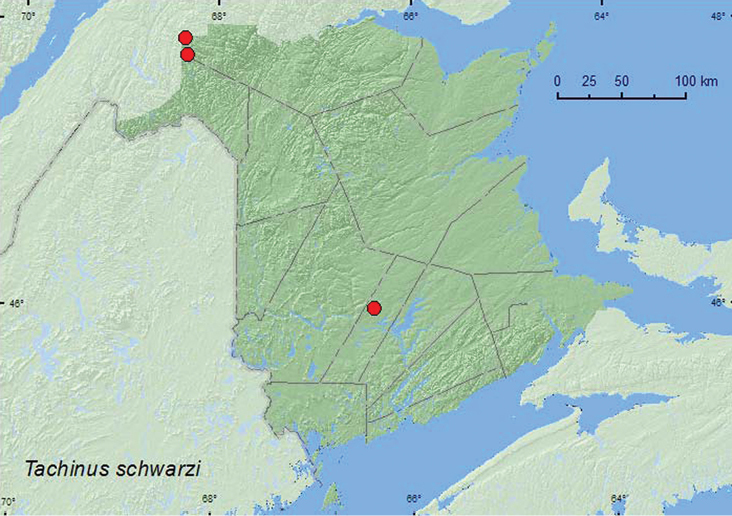
Collection localities in New Brunswick, Canada of *Tachinus schwarzi*.

##### Collection and habitat data.

Specimens of this species from New Brunswick were collected from a decaying fleshy polypore mushroom on a standing, dead spruce in a mature red spruce forest, from a decaying *Climacodon septentrionale* (Fr.) P. Karst. on a dead, standing yellow birch in a recent clearcut (boreal forest area), and from a (fresh) *Climacodon septentrionale* (Fr.) P. Karst. on a living sugar maple in an old-growth northern hardwood forest. Four individuals were collected in company with *Lordithon niger* (Gravenhorst) from a decaying fleshy polypore fungus on a standing, dead *Populus* sp. in a hardwood forest (sugar maple and American beech) in Saint-Raphaël (15.VII.2006), Quebec (Webster, unpublished). One specimen from Tennessee (USA) was sifted from leaf litter. Little was previously known about the habitat and biology of this rare species. Campbell (1973) suggested that this species lived in some restricted habitat, such as mammal burrows. The habitat data above suggest that this species may be associated with decaying fleshy polypore or polypore-like fungi on standing dead and living trees. Adults were collected during July and August.

##### Distribution in Canada and Alaska.

QC, **NB**, NS (Campbell 1973, 1988).

#### Tachinus vergatus

Campbell, 1973**

http://species-id.net/wiki/Tachinus_vergatus

Map 11

##### Material examined.

**New Brunswick, Queens Co.**, Cranberry Lake P.N.A, 46.1125°N, 65.6075°W, 21–28.VII.2009, 2.IX.2009, R. Webster & M.-A. Giguère, old red oak forest, Lindgren funnel traps (2, RWC). **Restigouche Co.**, Dionne Brook P.N.A. 47.9030°N, 68.3503°W, 14–28.VII.2011, M. Roy & V. Webster, old-growth northern hardwood forest, Lindgren funnel trap (1, RWC); same locality and collectors but 47.9064°N, 68.3441°W, 31.V-15.VI.2011, M. Roy & V. Webster, old-growth white spruce and balsam fir forest, Lindgren funnel trap (1, RWC). **Sunbury Co.**, Burton, SW of Sunpoke Lake, 45.7575°N, 66.5736°W, 16.IV.2005, R. P. Webster, red maple swamp, in leaf litter near margin of slow stream (1, RWC). **York Co.** Charters Settlement, 45.8340°N, 66.7450°W, 22.IV.2005, R. P. Webster, mature mixed forest, in wood pile under bark of spruce (3, RWC); same locality, collector and forest type but 45.8395°N, 66.7391°W, 23.IV.2008, mixed forest, in flight, collected with net between 15:00 and 18:00h (1, RWC); 15 km W of Tracy off Rt. 645, 45.6848°N, 66.8821°W, 22–25.IV.2009, 4–11.VIII.2009, R. Webster and M.-A. Giguère, old red pine forest, Lindgren funnel traps (2, AFC, RWC).

**Map 11.**
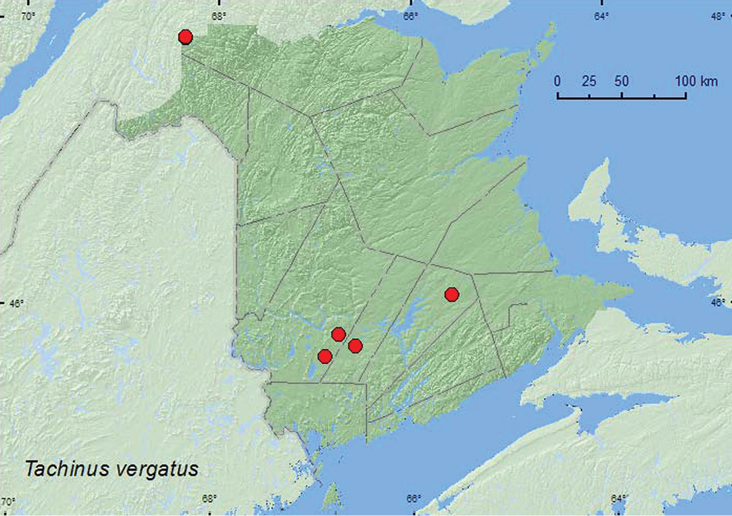
Collection localities in New Brunswick, Canada of *Tachinus vergatus*.

##### Collection and habitat data.

Little is known about the habitat associations of this species.Two adults of this rare species were collected from deciduous leaf litter along a small stream and from alder litter on a lake margin (Campbell 1975b). Others were taken from flight intercept traps (Campbell 1988). In New Brunswick, adults were collected from under bark in a wood pile, from leaf litter near a stream, and with a net during an evening flight. Some individuals were collected in Lindgren funnel traps deployed in old red oak forest, an old-growth red pine forest, an old-growth white spruce and balsam fir forest, and an old-growth northern hardwood forest. Adults were collected during April, June, July, August, and September but most during April.

##### Distribution in Canada and Alaska.

AB,ON, QC, **NB** (Campbell 1973, 1975b, 1988; Pohl et al. 2007).

#### Tachyporus lecontei

Campbell, 1991**

http://species-id.net/wiki/Tachyporus_lecontei

Map 12

##### Material examined.

**New Brunswick, Queens Co.**, just W of Jemseg at “Trout Creek”,45.8227°N, 66.1240°W, 9.V.2004, R. P. Webster, silver maple swamp, sifting leaf litter at base of large tree (3, NBM); same locality, forest type, and collector but 45.8231°N, 66.1245°W, 3.IV.2006, sifting litter from crotch of silver maple with multiple trunks (11, NBM, RWC); Grand Lake near Scotchtown, 45.8762°N, 66.1816°W, 25.IV.2004, R. Webster & M.-A. Giguère, oak/maple forest, under leaf litter at base of tree (1, NBM).

**Map 12.**
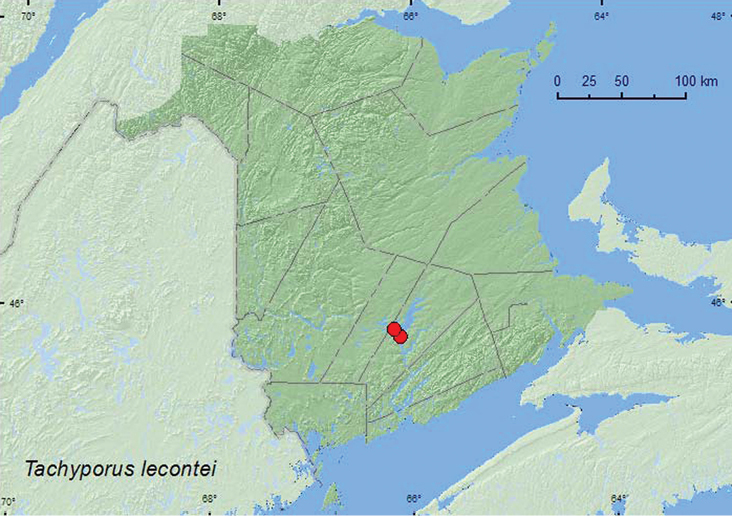
Collection localities in New Brunswick, Canada of *Tachinus lecontei*.

##### Collection and habitat data.

Campbell (1991) reported this species from river banks, flood debris on rivers, under logs, and decaying vegetation. Most New Brunswick specimens were found in litter in crotches of silver maples with multiple trunks in an old silver maple swamp (floodplain forest) early in April. This habitat was probably an overwintering site for this species. Many other staphylinid adults of various species were found in the debris in these tree crotches. Other individuals were sifted from leaf litter at the bases of large silver maples. Adults were collected during April and May.

##### Distribution in Canada and Alaska.

BC, AB, SK, MB, ON, QC, **NB** (Campbell 1991).

#### Tachyporus maculicollis

LeConte, 1866

http://species-id.net/wiki/Tachyporus_maculicollis

Map 13

##### Material examined.

**New Brunswick, Carleton Co.**, Two Mile Brook Fen, 46.3594°N, 67.6800°W, 2.VI.2005, R. P. Webster, *Care*x marsh, treading *Carex* hummock into water (1, RWC); Jackson Falls, Bell Forest, 46.2208°N, 67.7231°W 19.IV.2006, R. P. Webster, mature hardwood forest, in litter and moss near brook (2, RWC); same locality, forest type, and collector, 12.IV.2007, in leaf litter at base of tree, 30–40 cm of snow still on ground, (2, RWC); Meduxnekeag Valley Nature Preserve, 46.1888°N, 67.6762°W, 20.V.2005, M.-A. Giguère & R. Webster, river margin in flood debris (1, RWC). **Queens Co.**, just W of Jemseg at “Trout Creek”, 45.8231°N, 66.1245°W, 3.IV.2006, R. P. Webster, silver maple swamp, sifting litter from crotch of silver maple with multiple trunks (1, RWC). **Restigouche Co.**, near Little Tobique River, 47.4465°N, 67.0689°W, 24.V.2007, R. P. Webster, river margin, in leaf litter under alders (1, RWC); Jacquet River Gorge P.N.A., at Jacquet River, 47.8257°N, 66.0779°W, 24.V.2010, R. P. Webster, partially shaded cobblestone bar near mouth of brook, under cobblestones and gravel on sand (1, NBM). **York Co.**, Canterbury, near Browns Mountain Fen, 45.9033°N, 67.6260°W, 2.V.2005, R. P. Webster, red maple swamp, vernal pond margin in leaf litter (1, RWC); Charters Settlement, 45.8395°N, 66.7391°W, 5.IX.2006, R. P. Webster, mixed forest, among decaying (moldy) corncobs and cornhusks (1, RWC).

**Map 13.**
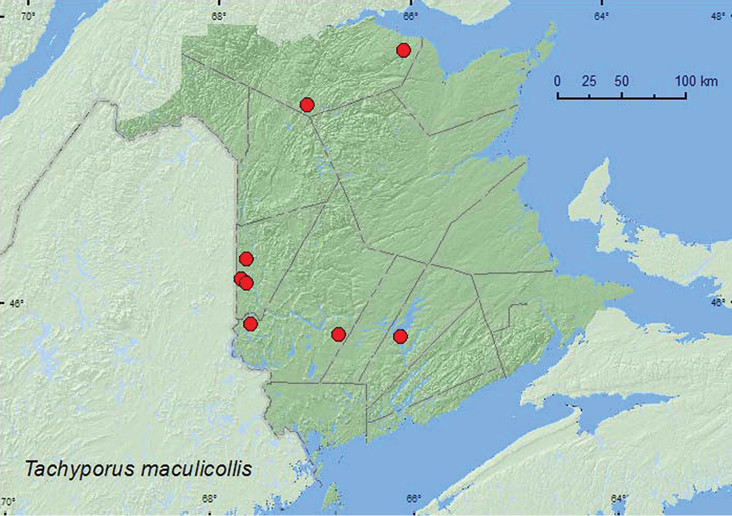
Collection localities in New Brunswick, Canada of *Tachinus maculicollis*.

##### Collection and habitat data.

Campbell (1991) reported this species from a variety of habitats, including forest leaf litter, fungi on an old tree stump, among grass roots, damp moss near a pond, under a log on a lakeshore, and from *Microtus pennsylvanicus *(Ord) nests. In New Brunswick, specimens were found in a various microhabitats including leaf litter and moss near brooks, margins of vernal ponds and a river, leaf litter at bases of trees, and among decaying corncobs and cornhusks. This species was found in a *Carex* marsh, mature hardwood forests, silver maple swamps, mixed forests, and river and brook margins. Adults were collected during April, May, June, and September and were active early in the spring on bare patches around bases of trees when over 30 cm of snow was still on the ground.

##### Distribution in Canada and Alaska.

BC, AB, SK, MB, ON, QC, **NB**, NS (Campbell 1979).

#### Tachyporus nanus

Erichson, 1839**

http://species-id.net/wiki/Tachyporus_nanus

Map 14

##### Material examined.

**New Brunswick, Sunbury Co.**, Acadia Research Forest, 45.9866°N, 66.3841°W, 19–25.V.2009, R. Webster & M.-A. Giguère, mature (110 year-old) red spruce forest with scattered red maple and balsam fir, Lindgren funnel traps (2 ♂, AFC, RWC). **York Co.**, 15 km W of Tracy, off Rt. 645, 45.6848°N, 66.8821°W, 19–25.V.2009, R. Webster & M.-A. Giguère, old (120–180 year-old) red pine forest, Lindgren funnel trap (1 ♂, RWC).

**Map 14.**
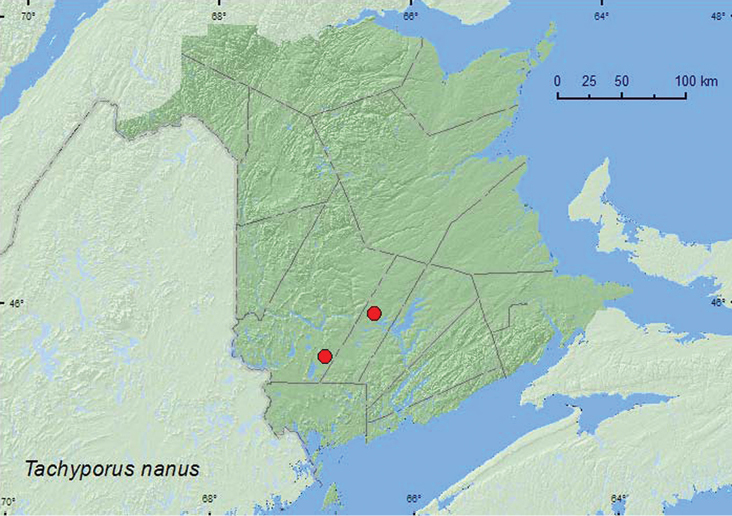
Collection localities in New Brunswick, Canada of *Tachinus nanus*.

##### Collection and habitat data.

This rare species has been collected from the fallen nest of a squirrel and a Berlese sample of decaying moldy material from the base of a tree (Campbell 1979). The three specimens from New Brunswick were captured in Lindgren funnel traps deployed in a 110-year-old red spruce forest and an old (120- to 180-year-old) red pine forest. Adults were collected during May.

##### Distribution in Canada and Alaska.

ON, QC, **NB** (Campbell 1979).

#### Tachyporus pulchrus

Blatchley, 1910**

http://species-id.net/wiki/Tachyporus_pulchrus

Map 15

##### Material examined.

**New Brunswick, Charlotte Co.**, 3.5 km NW of Pomeroy Ridge, 45.3087°N, 67.4362°W, 16.VI.2008, R. P. Webster, red maple swamp, in leaves and moss near small vernal pool (1, RWC). **Northumberland Co.,** Goodfellow Brook P.N.A., 46.8943°N, 65.3796°W, 23.V.2007, R. P. Webster, old growth wet eastern white cedar swamp, in grass litter and moss on hummocks near pool (1 ♀, NBM). **Sunbury Co.**, W of Sunpoke Lake, 45.7589°N, 66.5779°W, 22.IV.2006, R. P. Webster, red maple swamp, in moist leaves near vernal pool (1, NBM). **York Co.** Charters Settlement, 45.8267°N, 66.7343°W, 16.IV.2005, 9.IV.2006, 21.IV.2006, 23.V.2006, 14.IX.2006, R. P. Webster, *Carex* marsh/fen, in sphagnum hummocks (treading) and in leaf litter at bases of trees and shrubs (9, NBM, RWC); Mazerolle Settlement, 45.8729°N, 66.8311°W, 9.IV.2006, R. P. Webster, stream margin, in litter at base of eastern white cedar (2, NBM, RWC); off Hwy 2, N of Hanwell, 45.8987°N, 66.7903°W, 9.IV.2006, R. P. Webster, open grassy alder swamp, in grass litter (1, RWC); 9 km W of Tracy, 45.6888°N, 66.8004°W, 22.V.2008, R. P. Webster, *Carex* marsh/flowage, treading *Carex* hummock (1, NBM).

**Map 15.**
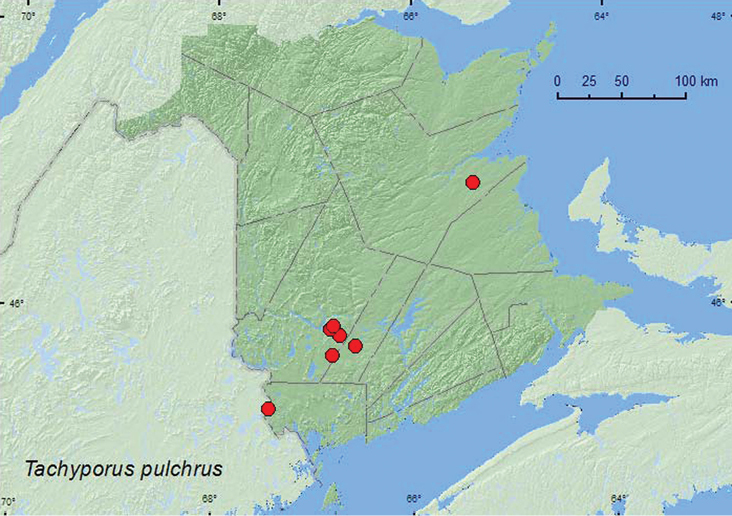
Collection localities in New Brunswick, Canada of *Tachinus pulchrus*.

##### Collection and habitat data.

This uncommon species was reported from dead swamp grass, among leaves, from moss, and from an entrance to a *Marmota* burrow by Campbell (1979). In New Brunswick, *Tachyporus pulchrus* was found in various wetland habitats, including eastern white cedar swamps, red maple swamps, an open grassy alder swamp, a *Carex* marsh/fen, a *Carex* marsh/flowage, and a stream margin near an eastern white cedar swamp. Adults occurred in leaves and moss, grass litter and moss on hummocks, sphagnum hummocks, leaf litter at bases of trees, and in grass litter and were collected by sifting or treading. This species was collected during April, May, June, and September (most during April).

##### Distribution in Canada and Alaska.

MB, ON**, NB** (Campbell 1979).

#### Tachyporus transversalis

Gravenhorst, 1806**

http://species-id.net/wiki/Tachyporus_transversalis

Map 16

##### Material examined. 

**New Brunswick, Carleton Co.**, Two Mile Brook Fen, 46.3619°N, 67.6733°W, 6.V.2005, R. P. Webster, eastern white cedar swamp, in litter at base of cedar (1, NBM); near Hovey Hill P.N.A., 46.1152°N, 67.7632°W, 10.V.2005, R. P. Webster, mixed forest with cedar, vernal pond margin, in moist leaves on muddy soil (4, RWC). **Charlotte Co.**, Rt. 3 at Deadwater Brook, 45.4744°N, 67.1225°W, 3.VI.2005, R. P. Webster, black spruce forest (forested bog) in moist sphagnum (1, RWC). **Saint John Co.**, Musquash, 45.1856°N, 66.3402°W, 30.V.2006, R. P. Webster, freshwater marsh, in litter on hummock (1, RWC). **York Co.**, Charters Settlement, 45.8267°N, 66.7343°W, 16.IX.2005, 29.III.2006, R. P. Webster, sedge fen, in litter and moss at base of tree (2, NBM, RWC); Mazerolle Settlement, 45.8788°N, 66.8311°W, 9.IV.2006, R. P. Webster, margin of stream in litter at base of cedar (1, RWC); 9 km W of Tracy off Rt. 645, 45.6888°N, 66.8004°W, 22.V.2008, R. P. Webster, sedge marsh, in *Carex* hummock (3, NBM, RWC); New Maryland, U.N.B. Woodlot, 45.9116°N, 66.6698°W, 26.V.2008, R. Webster, G. Forbes, & M.-A. Giguère, abandoned beaver lodge occupied by muskrats, in debris in roof of lodge (1, RWC).

**Map 16.**
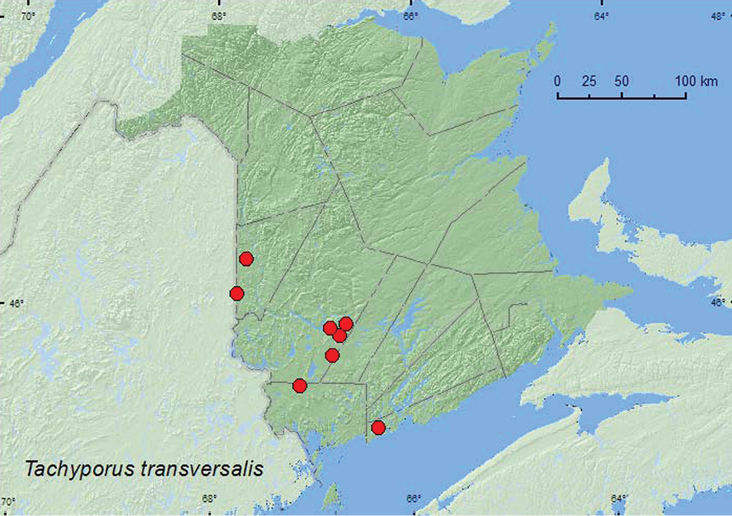
Collection localities in New Brunswick, Canada of *Tachinus transversalis*.

##### Collection and habitat data.

This is a hygrophilous species in both Europe and North America and is usually found in marshes and bogs in moss (especially sphagnum) and debris (Campbell 1991). In New Brunswick, this species was found in various wetland habitats, including a forested black spruce (*Picea mariana* (Mill.) B.S.P.) bog, an eastern white cedar swamp, sedge (*Carex*) fens and marshes, freshwater marshes, stream margins, the margin of a vernal pond, and in an abandoned North American beaver *Castor canadensis* Kuhl) lodge occupied by muskrats (*Ondatra zibethicus* L.). Adults occurred in moist leaves, sphagnum, litter, and moss, in *Carex* hummocks, and in debris in the roof of a beaver lodge. In New Brunswick, this species was collected during March, April, May, and June.

##### Distribution in Canada and Alaska.

ON, QC, **NB** (Campbell 1991). Campbell (1991) considered the distribution and habitat of this species in the Nearctic region unusual for a Holarctic species because of the specialized habitat preferences (found in sphagnum and debris in marshes and bogs) and pattern of distribution (restricted to Ottawa Valley of Ontario and Quebec). He suggested that the species may have been overlooked by most collectors and may actually have a broader distribution in North America than the records indicate. Klimaszewski et al. (2010) considered *Tachyporus transversalis* Gravenhorst as an adventive Palaearctic species.

### Tribe Mycetoporini Thomson, 1859

#### Ischnosoma flavicolle

(LeConte, 1863)**

http://species-id.net/wiki/Ischnosoma_flavicolle

Map 17

##### Material examined.

**CANADA, New Brunswick, Carleton Co.**, Jackson Falls, Bell Forest, 46.2208°N, 67.7231°W, 19.IV.2005, R. P. Webster, mature hardwood forest, in moss and litter near stream (1, RWC). **Charlotte Co.**, 3.0 km NW of Pomeroy Ridge, 45.3059°N, 67.4343°W, 5.VI.2008, R. P. Webster, alder swamp, in moss hummocks with grasses (1, RWC). **Queens Co.**, Upper Gagetown, bog adjacent to Hwy 2, 45.8316°N, 66.2346°W, 12.IV.2006, R. P. Webster, tamarack bog, in sphagnum hummock in open bog (2, NBM, RWC). **Saint John Co.,** Chance Harbour, off Rt. 790, 45.1355°N, 66.3672°W, 15.V.2006, R. P. Webster, calcareous fen, in sphagnum and litter among *Carex* (1, RWC). **Sunbury Co.**, Burton, SW of Sunpoke Lake, 45.7575°N, 66.5736°W, 10.IV.2005, R. P. Webster, red maple swamp, in leaf litter at base of tree (1, RWC). **York Co.** Charters Settlement, 45.8267°N, 66.7343°W, 9.IV.2005, 16.IV.2005, R. P. Webster, *Carex* marsh/fen, in sphagnum hummocks and litter at base of trees (6, NBM, RWC); same locality and collector but 45.8428°N, 66.7279°W, 20.IV.2005, small sedge marsh, in moist grass litter and sphagnum (1, RWC); 14 km WSW of Tracy, S of Rt. 645, 45.6755°N, 66.8685°W, 4.IX.2008, R. P. Webster, red maple swamp with alders, sifting moist leaf litter and moss (1, NBM).

**Map 17.**
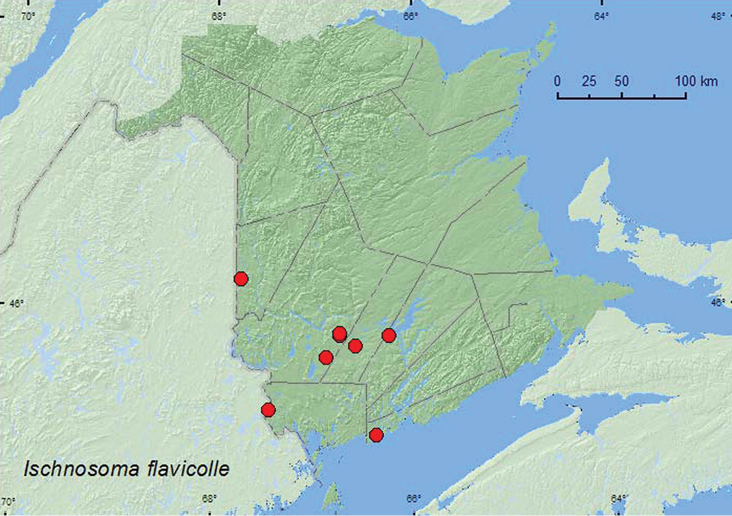
Collection localities in New Brunswick, Canada of *Ischnosoma flavicolle*.

##### Collection and habitat data.

Campbell (1991) reported that this species was often found in drier habitats than other members of the *Pictum* group of species. Adults were reported from various kinds of forest litter in pine, hardwood, and mixed pine and hardwood forests as well as cypress forests (Campbell 1991). In New Brunswick, this species was most often found in and near wetland habitats, such as calcareous fens, *Carex* marshes, tamarack (*Larix laricina* (Du Roi) Koch) bogs, alder swamps, and red maple swamps. One adult was found near a stream in a mature hardwood forest. Adults occurred in moss and sphagnum hummocks, moss, leaf and grass litter at bases of trees, and *Carex* hummocks. Adults were collected during April, May, June, and September.

##### Distribution in Canada and Alaska.

ON, **NB** (Brunke and Marshall 2011). In the United States, *Ischnosoma flavicolle* occurs throughout the southeast north to New Hampshire along the eastern seaboard (Campbell 1991).

#### Ischnosoma splendidum

(Gravenhorst, 1806)

http://species-id.net/wiki/Ischnosoma_splendidum

Map 18

##### Material examined.

**New Brunswick, Carleton Co.**, Meduxnekeag River Valley Nature Preserve, 46.1907°N, 67.6740°W, 7.IX.2004, R. P. Webster, small balsam fir stand (near hardwood stand), in fleshy gilled mushrooms (2, RWC); same locality, forest type and collector, 11.V.2005, in moldy conifer duff (4, RWC).

**Map 18.**
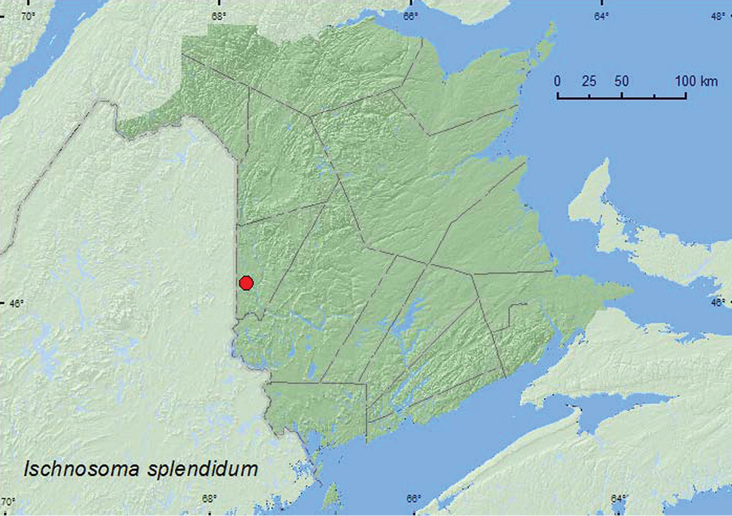
Collection localities in New Brunswick, Canada of *Ischnosoma splendidum*.

##### Collection and habitat data.

Campbell (1991) reported this species from various of wetland habitats as well as forests. Adults were taken from flood debris along rivers, margins of beaver ponds, beaver lodges, muskrat nests, mallard (*Anas platyrhynchos* L.) nests, moss near seepage areas, leaf litter along margins of marshes, streams, and bogs, and various kinds of grass and leaf litter from conifer and deciduous forests (Campbell 1991). Recent studies in Alberta (mid-boreal ecoregion) by Buddle et al. (2006) indicated that this species was associated with old (>70-year-old) fire-origin, mixed wood forest stands. Later, Pohl et al. (2007) reported that this species was also associated with regenerating mixed wood stands in the western foothills of Alberta. The specimens from New Brunswick were collected in moldy conifer duff and fleshy gilled mushrooms in a small balsam fir (*Abies balsamea* (L.) Mill.) (regenerating) stand adjacent to a hardwood forest. Campbell (1991) reported most specimens of this species were taken in June to September. The specimens from New Brunswick were collected during May and September.

##### Distribution in Canada and Alaska.

AK, YT, NT, BC, AB, SK, MB, ON, QC, **NB**, NS, LB, NF (Campbell 1991).

#### Lordithon (Bolitobus) longiceps

(LeConte, 1863)

http://species-id.net/wiki/Lordithon_longiceps

Map 19

##### Material examined.

**New Brunswick, Carleton Co.**, Jackson Falls, Bell Forest, 46.2200°N, 67.7231°W, 16.IX.2006, R. P. Webster, mature hardwood forest, on *Bjerkandera adusta* (Willd.) P. Karsten on dead standing beech tree and on a beech log (2 ♂, 4 ♀, RWC); Meduxnekeag River Valley Nature Preserve, 46.1897°N, 67.6710°W, 12.IX.2008, R. P. Webster, mixed forest, on mass of *Pholiota* sp. mushrooms at base of dead standing *Populus* sp. (1 ♂, RWC). **Restigouche, Co.**, Dionne Brook P.N.A., 47.9064°N, 68.3441°W, 31.V-15.VI.2011, K. Van Rooyen & C. Hughes, old-growth white spruce and balsam fir forest, Lindgren funnel trap (1 ♀, RWC).

**Map 19.**
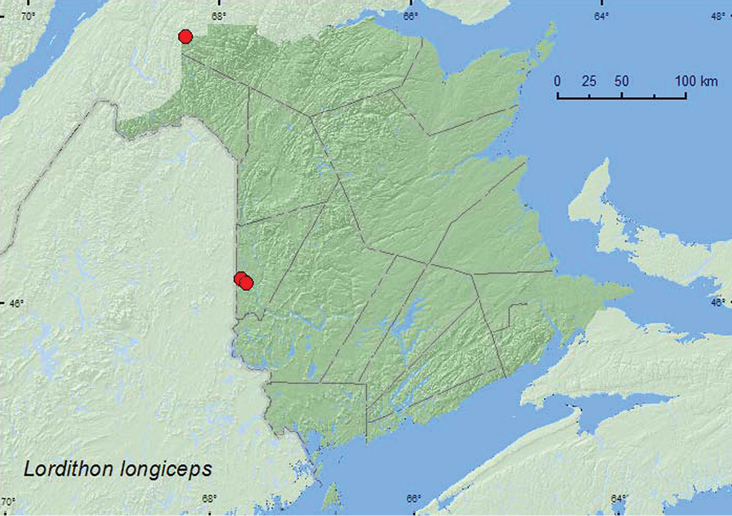
Collection localities in New Brunswick, Canada of *Lordithon longiceps*.

##### Collection and habitat data.

Little is known about the habitat requirements of this rare species. Campbell (1982) reported that adults have been taken on rotting gilled mushrooms. Most of the specimens from New Brunswick were collected from the fleshy polypore fungus, *Bjerkandera adusta* (Willd.) P. Karsten growing on a dead, standing American beech tree and a beech log. One individual was found in a mass of *Pholiota* sp. mushrooms at the base of a dead, standing *Populus* sp. Another individual was captured in a Lindgren funnel trap. Adults were found in mature hardwood and adjacent mixed forests, and in an old-growth white spruce and balsam fir forest. This species was collected during September in New Brunswick.

##### Distribution in Canada and Alaska.

AK, BC, AB, ON, PQ, **NB**, NS (Campbell 1982; Campbell and Davies 1991). This northern species has a very broad distribution from Alaska to Nova Scotia, but with large distributional gaps between known localities (Campbell 1982).

#### Lordithon (Bolitobus) quaesitor

(Horn, 1877)

http://species-id.net/wiki/Lordithon_quaesitor

Map 20

##### Material examined.

**New Brunswick, Albert Co.**, Caledonia Gorge P.N.A., near Turtle Creek, 45.8380°N, 64.8484°W, 3.VII.2011, A. Fairweather & R. P. Webster, old-growth sugar maple and yellow birch forest, on *Polyporus varius* (1 ♂, 1 ♀, RWC); same locality but 45.8415°N, 64.8467°W, 5.VII.2011, R. P. Webster, old-growth sugar maple and yellow birch forest, on *Polyporus varius* on dead standing beech (1 ♂, NBM). **Carleton Co.**, Meduxnekeag River Valley Nature Preserve, 46.1907°N, 67.6740°W, 4.VIII.2006, R. P. Webster, mature hardwood forest,in *Bjerkandera adusta* (Willd.) P. Karsten on side of beech log (1 ♀, RWC); Meduxnekeag River Valley Nature Preserve, 46.1878°N, 67.6705°W, 2.IX.2008, R. P. Webster, hardwood forest,in *Bjerkandera adusta* (Willd.) P. Karsten on side of beech log (2 ♀, NBM, RWC); same locality and collector, 2.IX.2008, hardwood forest, on *Pleurotus* sp. mushroom on side of log (1 ♀, NBM); Jackson Falls, Bell Forest, 46.2200°N, 67.7231°W, 16.IX.2006, R. P. Webster, mature hardwood forest, on *Bjerkandera adusta* (Willd.) P. Karsten on dead standing beech tree and on beech log (3 ♀, RWC). **Restigouche Co.**, Dionne Brook P.N.A., 47.9030°N, 68.3503°W, 19.IX.2011, R. P. Webster, old-growth northern hardwood forest, in gilled mushroom (1, RWC). **Sunbury Co.**, Acadia Research Forest, 45.9866°N, 66.3841°W, 9–16.VI.2009, R. Webster & M.-A. Giguère, mature (110 year-old) red spruce forest with scattered red maple and balsam fir, Lindgren funnel trap (1, AFC). **York Co.** Charters Settlement, 45.8340°N, 66.7450°W, 20.V.2007, R. P. Webster, mixed forest, in polypore fungi on *Populus* sp. log (1 ♀, RWC); Kelly’s Creek at Sears Rd., 45.8723°N, 66.8414°W, 8.VI.2008, R. P. Webster, alder swamp, on *Pleurotus* sp. on dead standing balsam poplar (1 ♀, RWC).

**Map 20.**
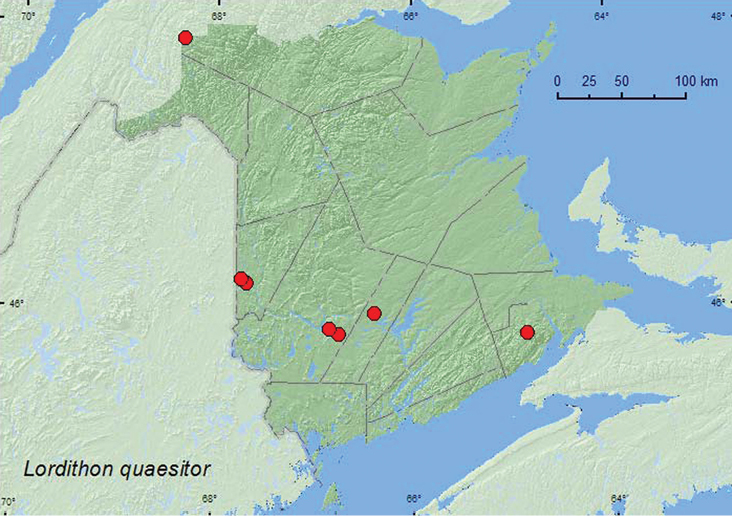
Collection localities in New Brunswick, Canada of *Lordithon quaesitor*.

##### Collection and habitat data.

Little was previously known about the habitat associations of this rare species (Campbell 1982). In New Brunswick, adults of *Lordithon quaesitor* were most frequently found on *Bjerkandera adusta* (Willd.) P. Karsten (a fleshy polypore fungus) on the side of beech logs and standing dead beech trees in mature hardwood forests. This species was also found in *Pleurotus* sp., among a group of *Polyporus varius* Fr. on a large sugar maple log and on a dead standing American beech in an old-growth hardwood forest with sugar maple and yellow birch, in polypore fungi on sides of logs, in *Pleurotus* sp. on a dead standing balsam poplar (*Populus balsamifera* L.) tree, and in a gilled mushroom on the forest floor of an old-growth northern hardwood forest with sugar maple and yellow birch. One adult was collected in a Lindgren funnel trap. Some adults were collected in a mature red spruce forest, a mixed forest, and an alder swamp adjacent to a mixed forest. *Lordithon quaesitor* was sometimes found together with *Lordithon niger*, *Lordithon axillaris*, and *Lordithon longiceps* and probably has a similar biology to those species. Adults were collected during May, June, July, August, and September.

##### Distribution in Canada and Alaska.

ON, QC, **NB**, NS (Campbell 1976; Campbell and Davies 1991; Bishop et al. 2009).

#### Lordithon (Lordithon) axillaris

(Gravenhorst, 1806)**

http://species-id.net/wiki/Lordithon_axillaris

Map 21

##### Material examined.

**New Brunswick, Carleton Co.**, Hovey Hill P.N.A., 46.1115°N, 67.7770°W, 19.VIII.2004, R. P. Webster, mature hardwood forest, on *Pleurotus* sp. on side of log (1 ♂, 1 ♀, RWC); Meduxnekeag River Valley Nature Preserve, 46.1940°N, 67.6800°W, 23.VI.2006, 3.VII.2006, R. P. Webster, mixed forest, on *Pleurotus* sp on dead standing *Populus* sp. (2 ♂, RWC); Meduxnekeag River Valley Nature Preserve, 46.1878°N, 67.6705°W, 18.VIII.2008, R. P. Webster, hardwood forest,in large (orange) gilled mushrooms near base of dead standing beech tree (2 ♂, 3 ♀, RWC, NBM); same locality but 46.1887°N, 67.6735°W, 18.VI.2010, R. P. Webster, hardwood forest, in *Laetiporus sulphureus* (1, RWC).

**Map 21.**
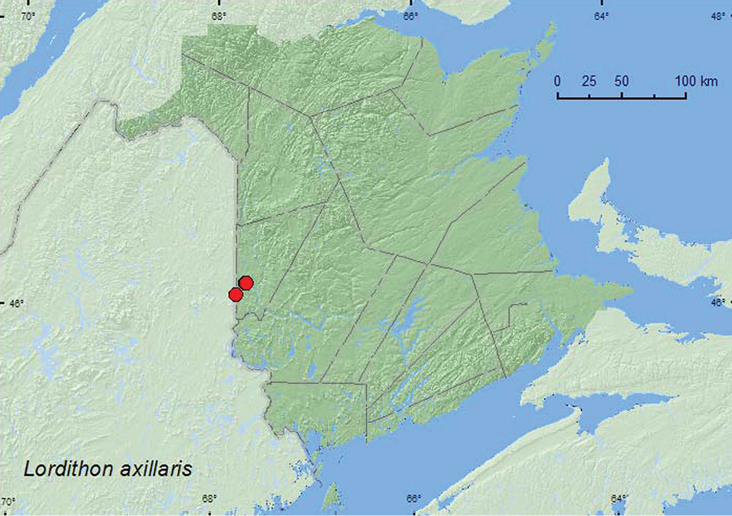
Collection localities in New Brunswick, Canada of *Lordithon axillaris*.

##### Collection and habitat data.

Little was previously known about the habitat associations of this rare species. One specimen from Quebec was collected from large gilled mushrooms on the side of a log (Campbell 1982). Four specimens of this species were collected from *Pleurotus* sp. mushrooms on the side of *Populus* logs in a hardwood forest in Saint-Raphaël (15.VII.2006), Quebec (Webster, unpublished). Most specimens from New Brunswick were collected from *Pleurotus* sp. mushrooms on standing dead *Populus* sp. trees or on the side of logs in mature hardwood forests with sugar maple and beech. Some adults were also collected from a large orange-gilled mushroom on the side of a log. One individual was collected from inside a *Laetiporus sulphureus* (Fr.) Murr. (Polyporaceae). These data suggest that this species may be specialized on *Pleurotus* sp. and other large gilled mushrooms that grow on standing dead trees or logs. Campbell (1982) suggested that this species, like *Lordithon niger* and the European *Lordithon bicolor* (Gravenhorst), may be associated with old-growth hardwood forests. Adults of *Lordithon axillaris* were collected during June, July, and August in New Brunswick.

##### Distribution in Canada and Alaska.

QC, **NB** (Campbell 1982).

#### Lordithon (Lordithon) campbelli

Schülke, 2000***

http://species-id.net/wiki/Lordithon_campbelli

Map 22

##### Material examined.

**CANADA, New Brunswick, Carleton Co.**, Meduxnekeag River Valley Nature Preserve, 46.1940°N, 67.6800°W, 23.VI.2006, R. P. Webster, mixed forest, on *Pleurotus* sp on dead standing *Populus* sp. (1 ♀, RWC); Jackson Falls, Bell Forest, 46.2200°N, 67.7231°W, 19.VII.2006, R. P. Webster, mature hardwood forest, in gilled mushroom (4 ♂, 3 ♀, RWC).

**Map 22.**
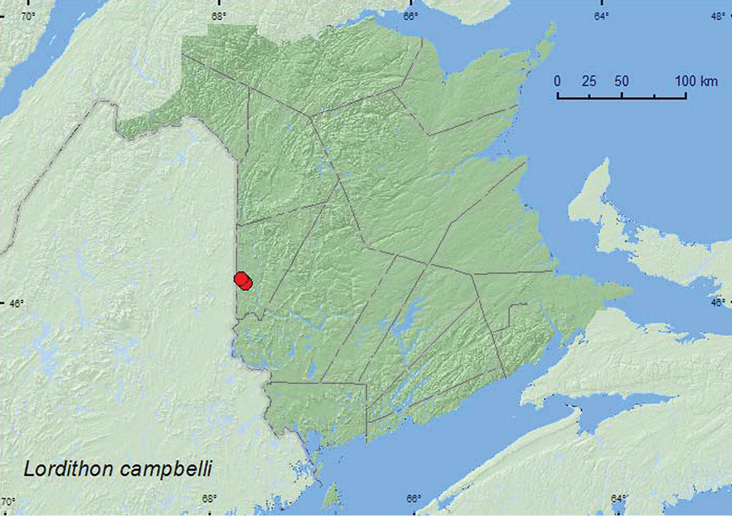
Collection localities in New Brunswick, Canada of *Lordithon campbelli*.

##### Collection and habitat data.

Campbell (1982) reported collecting adults from various gilled and pore mushroom species. In New Brunswick, adults were collected from gilled mushrooms on forest floor and on *Pleurotus* sp. on a dead, standing *Populus* sp. Adults were collected during June and July.

##### Distribution in Canada and Alaska.

**NB** (first Canadian record). In the United States, this species (as *Lordithon angularis* (Saches) in Campbell 1982) is distributed from Massachusetts to Florida, west to Missouri (Campbell 1982). This species probably occurs in intervening areas between Massachusetts and New Brunswick.

#### Lordithon (Lordithon) niger

(Gravenhorst, 1802)**

http://species-id.net/wiki/Lordithon_niger

Map 23

##### Material examined.

**New Brunswick, Carleton Co.**, Meduxnekeag River Valley Nature Preserve, 46.1907°N, 67.6740°W, 4.VIII.2006, R. P. Webster, mature hardwood forest,in *Bjerkandera adusta* (Willd.) P. Karsten (a fleshy polypore fungi) on side of beech log (1 ♀, RWC); Jackson Falls, Bell Forest, 46.2200°N, 67.7231°W, 16.IX.2006, R. P. Webster, mature hardwood forest, in *Bjerkandera adusta* (Willd.) P. Karsten on dead standing beech tree (1 ♀, RWC); same locality, collector, and forest type but 18.VIII.2008, in *Porodaedalea* sp. (fleshy polypore) on dead standing beech tree (1 ♂, RWC). **Queens Co.**, Cranberry Lake P.N.A, 46.1125°N, 65.6075°W, 11–18.VI.2009, R. Webster & M.-A. Giguère, old red oak forest, Lindgren funnel trap (1, AFC).

**Map 23.**
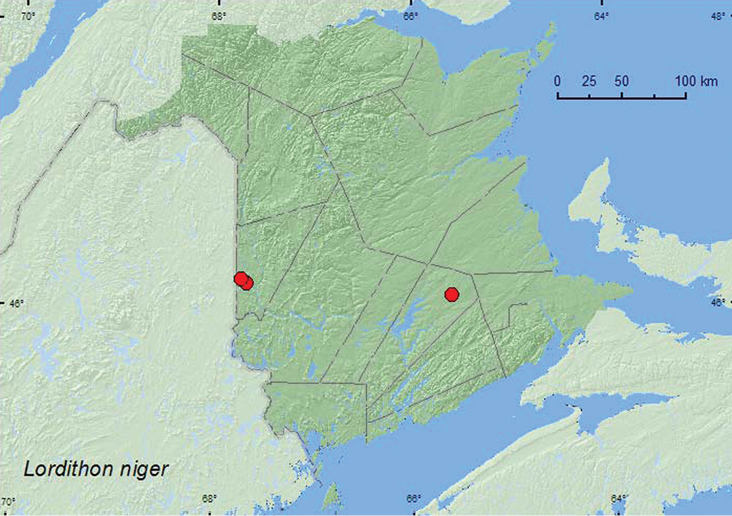
Collection localities in New Brunswick, Canada of *Lordithon niger*.

##### Collection and habitat data.

Nothing was previously known about the habitat associations of this rare species (Campbell 1982). The New Brunswick specimens were collected from *Bjerkandera adusta* (Willd.) P. Karsten and *Porodaedalea* sp. (both are fleshy polypore fungi) on a beech log, or on dead, standing beech trees in a mature to old-growth and predominantly hardwood forest. One individual was captured in a Lindgren funnel trap in a mature to old red oak forest. Several specimens of this species were found in company with *Tachinus schwarzi* in a decaying fleshy polypore fungus on a standing, dead *Populus* sp. in a hardwood forest in Saint-Raphaël (15.VII.2006), Quebec (Webster, unpublished). Adults from New Brunswick were collected during June, August, and September. The habitat data above suggest that this species might be specialized on fleshy polypore fungi and related species that grow on dead standing trees or logs. Campbell (1982) noted that this species appeared to be becoming increasingly rare and suggested that it might be associated with old-growth hardwood forests, which are disappearing from most of eastern North America. However, more sampling should be done in forests of various ages to establish if this species is indeed an old–growth associate.

##### Distribution in Canada and Alaska.

ON, QC, **NB** (Campbell 1982).

#### Mycetoporus americanus

Erichson, 1839**

http://species-id.net/wiki/Mycetoporus_americanus

Map 24

##### Material examined.

**New Brunswick, Restigouche Co.**, Berry Brook P.N.A., 47.8140°N, 66.7578°W, 26.V.2007, R. P. Webster, old growth eastern white cedar swamp, in moss on hummock at base of tree (1, RWC).

**Map 24.**
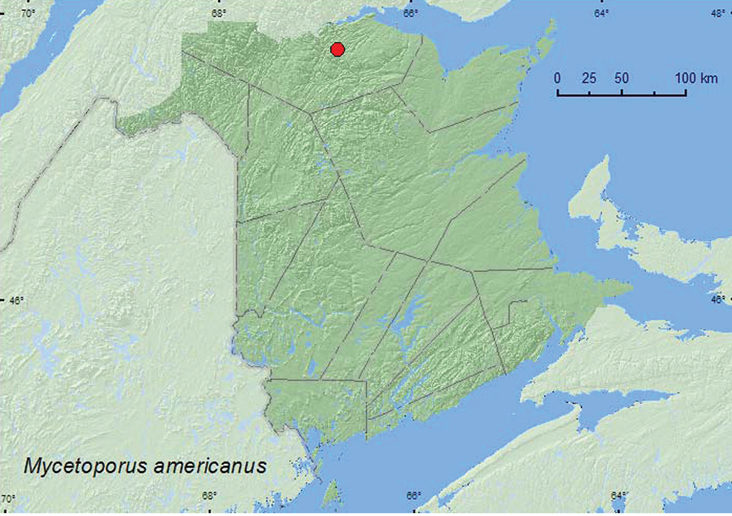
Collection localities in New Brunswick, Canada of *Mycetoporus americanus*.

##### Collection and habitat data.

Campbell reported that most adults of this species were collected along stream and lake margins. In Alberta, this species was associated with mature forests (Pohl et al. (2007). The New Brunswick specimen was collected from moss on a hummock at the base of a tree in an old-growth eastern white cedar swamp during May.

##### Distribution in Canada and Alaska.

AK, YT, AB, BC, SK, ON, QC, **NB**, LB, NF (Campbell 1991).

#### Mycetoporus rugosus

Hatch, 1957

http://species-id.net/wiki/Mycetoporus_rugosus

Map 25

##### Material examined.

**New Brunswick, Queens Co.**, Grand Lake near Scotchtown, 45.8762°N, 66.1816°W, 30.IV2006, R. P. Webster, oak and maple forest in leaves at base of oak (1, RWC); same locality and collector, 25.V.2006, lakeshore, in drift material (1, RWC). **Charlotte Co.**, Rt. 3 at Deadwater Brook, 45.4745°N, 67.1225°W, 23.IV.2006, R. P. Webster, black spruce forest, in sphagnum (1, RWC); 3.0 km NW of Pomeroy Ridge, 45.3059°N, 67.4343°W, 16.VI.2008, R. P. Webster, old growth eastern white cedar swamp, in leaves and moss near small vernal pool (1, RWC). **Northumberland Co.**, 12 km SSE of Upper Napan, 46.8991°N, 65.3682°W, 7.VI.2006, R. P. Webster, old growth eastern white cedar swamp, in moss and leaf litter (1, RWC). **Restigouche Co.**, Little Tobique River near Red Brook, 47.4462°N, 67.0689°W, 24.V.2007, R. P. Webster, old growth eastern white cedar swamp, in moss and leaf litter near brook (1, RWC); NE of confluence of Little Tobique River and Red Brook, 47.4501°N, 67.0577°W, 24.V.2007, R. P. Webster, old growth eastern white cedar swamp, in sphagnum (1, RWC);MacFarlane Brook P.N.A., 47.6018°N, 67.6263°W, 25.V.2007, R. P. Webster, old growth eastern white cedar swamp, in moss near brook (1, RWC).** Saint John Co.**, ca. 2 km NE of Maces Bay, 45.1161°N, 66.4560°W, 8.V.2006, R. P. Webster, eastern white cedar swamp, in sphagnum near brook (1, RWC).

**Map 25.**
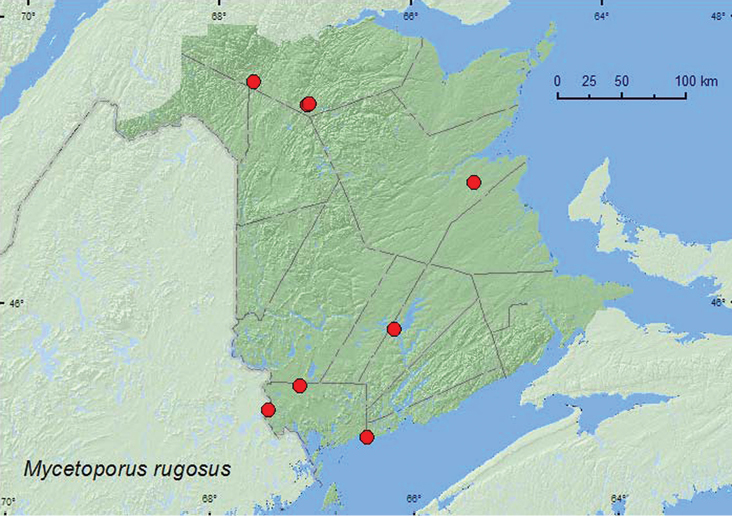
Collection localities in New Brunswick, Canada of *Mycetoporus rugosus*.

##### Collection and habitat data.

Adults of *Mycetoporus rugosus* have been collected from a wide variety of moist (often deep and moldy) litter and moss, including both deciduous and conifer litter of various species in forested habitats, as well as lake, stream, and river margins (Campbell 1991). New Brunswick specimens were collected from similar habitats, most frequently from moss and litter near brooks in old-growth eastern white cedar swamps. Adults were also collected from drift material and oak leaf litter along a lakeshore and from sphagnum in a black spruce forest. Adults from New Brunswick were collected during May and June.

##### Distribution in Canada and Alaska.

AK, NT, YT, BC, AB, SK, MB, ON, QC, **NB**, NS, LB (Campbell 1991)

## Supplementary Material

XML Treatment for Nitidotachinus horni

XML Treatment for Sepedophilus cinctulus

XML Treatment for Sepedophilus crassus

XML Treatment for Sepedophilus occultus

XML Treatment for Sepedophilus versicolor

XML Treatment for Tachinus addendus

XML Treatment for Tachinus canadensis

XML Treatment for Tachinus fimbriatus

XML Treatment for Tachinus frigidus

XML Treatment for Tachinus schwarzi

XML Treatment for Tachinus vergatus

XML Treatment for Tachyporus lecontei

XML Treatment for Tachyporus maculicollis

XML Treatment for Tachyporus nanus

XML Treatment for Tachyporus pulchrus

XML Treatment for Tachyporus transversalis

XML Treatment for Ischnosoma flavicolle

XML Treatment for Ischnosoma splendidum

XML Treatment for Lordithon (Bolitobus) longiceps

XML Treatment for Lordithon (Bolitobus) quaesitor

XML Treatment for Lordithon (Lordithon) axillaris

XML Treatment for Lordithon (Lordithon) campbelli

XML Treatment for Lordithon (Lordithon) niger

XML Treatment for Mycetoporus americanus

XML Treatment for Mycetoporus rugosus
